# Clinical Notes

**Published:** 1874-08-01

**Authors:** F. K. Bailey

**Affiliations:** Knoxville, Tenn.


MEDICAL EXAMINER.

A

*Semi-Monthly Journal of Medical Sciences.*

EDITED BY N. S. DAVIS, M.D., AND F. H. DAVIS, M.D.

No. XV. CHICAGO, AUG. 1, 1874. VOL. XV.

### Original Communications.

### CLINICAL NOTES.

By F. K. Bailey, M. D., Knoxville, Tenn.

RHEUMATIC FEVER. — Case I.—Miss M., aged twenty years; of light complexion and hair; sanguineo-nervous temperament (the nervous, perhaps, predominating); had not been in good health since the age of fifteen, and was inclined to epileptiform seizures. On the fifth of May, I found the pulse small and compressible, beating 108 times in the minute, and the cardiac sounds normal; an anæmic thrill, however, indicated poverty of blood and irritable heart; her tongue was slightly coated and white, but moist; she complained of epigastric oppression, anorexia and thirst for acid drinks, with occasional nausea; the alimentary canal was in a normal condition and the urine not scanty, but occasionally red, and voided with intermittent contraction of the bladder; severe headache extended over the forehead and toward the vertex; the feet, ankles, and legs nearly to the knees were slightly swollen, very sensitive to the touch, and intolerant of motion; the skin presented a mottled, purplish appearance, which was unaffected by pressure with the finger; a deep-seated pain, as if along the principal nerves, with a general burning sensation, affected the whole limb; there was well-marked objective heat, but the subjective sensations were those of occasional chilliness, general restlessness and inability to sleep.

I ordered a liniment to be applied on flannel to the affected limbs, containing half an ounce of chloroform and glycerine, and an ounce of the tincture of camphor, which, by 6 P. M., had produced much relief. The skin of the patient seemed of a natural hue, but considerable fever remained, with increased cephalalgia. I therefore directed her to take two grains of quinine every three hours during the night, and applied the liniment to the pit of the stomach and forehead, with marked temporary relief.

She rested well during the succeeding night, and I found in the morning that she had had no fever, and that the pulse was soft, full, and beating 100 times per minute. The limbs were less painful, and an enema had induced a moderate stool. The quinia was continued and lemon-juice ordered, while solid aliment was forbidden in consequence of the anorexia. No fever existed during the day, but slight cinchonism had occurred from taking sixteen grains of quinine. Her pulse was full—108 beats. I suspended the quinia and continued the embrocation.

She slept but little during the next night and complained of pain in the limbs, and about the hip joints and pelvic region, with nausea, feeling of faintness, and great desire for acids. I gave her ten drops of aromatic sulphuric acid in one drachm of compound tincture of gentian every three hours. At 6 P. M. there had been no fever; she was generally more comfortable, but could not take the medicine last ordered. Chicken soup was given every two or three hours during the night, and the liniment was used freely without further medication. Her limbs were much more swollen and painful on the next day, and she had had but little sleep. I ordered two and a half grains of the iodide of potassium and one twenty-fourth of a grain of sulphate of morphine to be given in pills every two hours, and substituted tincture of arnica for camphor in the liniment, which was still to be applied freely. Raw oysters were supplied as food. On Friday at 8 A. M. the patient was very much better and without pain, though a little soreness remained in her limbs. She had slept one-half of the night, and was faint at one time when port wine in small quantities procured relief. She had also had two or three severe paroxysms of hiccough and occasional epistaxis—a symptom which had occurred several times daily since I first saw her; her pulse was ninety-six and very soft; her tongue cleaner, and there was more desire for food and lemons; the latter produced no gastric uneasiness; the wine and food were continued freely and the pills given every four hours. On Saturday, at 9 A. M., she was suffering but little and had rested well; an enema had been followed by a stool; her pulse was eighty-five; her tongue cleaning, and the urine satisfactory. During the night she slept some, but was disturbed by frightful dreams; anorexia, cephalalgia, and mental depression remained, with sobbing and a "terrible feeling." I enjoined perfect rest and the ingestion of but little food, with small quantities of wine. At 6 P. M. she had slept nearly all day, and complained of soreness in the hips and inguinal region; her bowels moved spontaneously; her pulse was quite full, but soft, and about ninety; her tongue cleaning, and showing a smooth, red appearance in the middle, which is not unusual in nervous fevers. One pill was ordered at bed-time and beef tea occasionally during the night, with continued use of the embrocation. On Sunday, at 9 A. M., I found she had slept during the night, and had more desire for food; she said that she began to feel better, but toward morning she had the "petit mal;" the bromide of potassium was given with a pill of the oxyd of silver. On Tuesday, the 13th, she stated that she had sat up four hours during the previous afternoon and had slept well during the night; her appetite was returning; there had been a motion of the bowels and the white coat was disappearing from the tongue, except at the edges; still a raw beefy surface could be seen in the centre; her pulse was full and about ninety; her countenance showed less anxiety, and she complained but little of soreness in the limbs. On Wednesday, at 9 P. M., there was less pain than usual; her pulse was ninety, and soft, but tolerable full; she had slept well and could rise from bed unaided; her bowels moved once, from an enema, and her tongue was nearly free from the white coat, but still very red. On the 15th, at 1 P. M., I found she had slept during the night, but had had more pain and soreness in the muscles since morning; there was left some facial pain from a carious tooth; there was also thirst, increased heat of surface and a pulse beating 100 per minute; the potass pills and the other remedies were resumed. On the 20th, there was less muscular pain, but considerable suffering from soreness of the gums and edge of the tongue on the left side, and about the third molar tooth of the inferior maxilla; her tongue was covered with a thick white coat; her pulse beat at about eighty per minute, but was soft and regular; indifferent appetite for a few days had yielded to a desire for eggs and beef tea, which she seemed to relish; with the exception of extreme tenderness of the mouth she seemed quite convalescent; two of the pills at intervals of two hours produced sleep; her stools were normal; one grain of the sulphate of quinine was given every three hours, and the bromide of potassium and oxyd of silver continued. She had a good night on the 21st, and complained of no pain except in the jaw; liquid food was administered, as she was unable to masticate anything solid; the bromide of potassium and quinia were now suspended. She slept on the night of the 22d, but became much nauseated about 10, complaining of extreme faintness; her tongue became flabby and exhibited no coat, but the redness had disappeared; her pulse was feeble and slow; but little nutriment had been taken; chloroform liniment was applied to the pit of the stomach, and a few drops of chloroform were followed by small quantities of brandy with milk, internally. Two grains of the citrate of iron were ordered three times daily, and she soon became easier. After some sleep she felt better than she had for two or three days. The bowels moved quite freely on the night of the 22d, from an enema. She slept well on the night of the 27th; her tongue assumed a more natural hue and her pulse was normal in frequency and force, except that it was softer than in health; her bowels moved spontaneously and there was some return of appetite; she took brandy and milk, with fruit and toast, for two days, and was driven out on the 24th, feeling much refreshed afterward; the citrate of iron, which had thus far been tolerated, was continued, and also the oxyd of silver; there were some indications of the menstrual molimen during a part of the last week; it was the regular time for its recurrence, but for obvious reasons it might be deferred to any date before the end of the ensuing lunar month. On the 28th, she was much improved, and could sit up for a part of the day; the citrate of iron, pills and oxyd of silver were continued, with a caution as to exercise. Further visits were discontinued.

February, 1874.—This young lady has been subject, for some years, to an affection which was variously diagnosed by different medical men, but it is probable that the paroxysms were reflex in their origin, the uterine function being at fault. The physician under whose charge she had been for a year or more, prescribed bromide of potassium and bromide of ammonium, with oxyd of silver. The paroxysms were evidently epileptiform, and it was thought she had been improving for some time before this illness occurred.

The spasms occurred, generally, during sleep, and were so short that even the family seldom had a full opportunity of observing their commencement. Still, though she had been thus affected for four or five years, no evidence of impaired memory or any mental faculty, was evident. After convalescence had become established, I learned that the menses came on a few days before I was summoned, but were suspended after a few hours, in consequence, as she said, of her sitting down upon a cold floor while assisting in putting down a carpet. "Everything stopped suddenly." This circumstance, undoubtedly, was a principal cause of her sickness, or certainly, of the "nervous" phenomena. The pain and soreness of the limbs indicated a rheumatic condition, sometimes denominated muscular, and known, a few years ago, as rheumatalgia. The mottled hue noticed in the skin of the lower limbs, was suggestive of purpura, and I was reminded of this case, while reading, lately, in the *Compendium of Medical Science*, (Jan., 1874,) the details of a case given by Dr. Louis A. Duhring, of Philadelphia. Such may, perhaps, be properly classed with the "purpura febrilis simplex," of Wilson.

It is difficult, however, to give names to the different affections encountered in daily practice, and we must recognize pathological conditions, rather than any system of nomenclature which may be adopted.

The menses did not recur until about eight weeks after the time of attack; and it is proper to note, in this connection, that much of the sickness of young females, as well as those of riper years, is due to inattention during, and immediately preceding, the catamenial flow. American women are truly careless of their health. Some may attribute this to want of care on the part of mothers, but is it not true that mothers themselves are careless of their own health, and thus offer a bad example to their daughters? "Died of thin shoes and insufficient dressing," might be the verdict in thousands of cases all over our land. Women are too prone to walk the streets at all seasons, with a covering for the feet which no man, however unwise he may be in other respects, would think of wearing.

Case II. I visited with Dr. D. T. Boynton, a young man aged about thirty, of bilio-nervous temperament.

He had had, for some weeks, a fever attended with rheumatic pains in the knees, left shoulder and middle of the arm, but no noticeable pricking or numbness in the hand or fingers. His medical attendant stated that the pulse has not been over 100 more than two or three times. His bowels were irregular; urine red, but free, and, at times, copious.

His pulse was 100 when first counted, but soon diminished in frequency to 84. His tongue was slightly furred at the base, but from the middle to the tip it was of a bright red color, and smooth and moist in the whole extent. He suffered severely at night with pain in all the affected joints, as well as in the left side of the chest. He was somewhat thin in flesh, with rather an anxious countenance, and had taken iodide of potassium with bicarbonate of potassa laxatives, and deodorized tincture of opium. For a day or two he had also taken tincture of guiacum in small doses. I suggested the application of belladonna plaster to the cardiac region, if the pain should continue. The cardiac impulse had been considerably increased, but both sounds were distinct. The heart-beats were plainly audible as high as the second rib. There was no cardiac enlargement.

On the 13th of June, this young man was still unable to go out of doors, and suffered much from arthritic and muscular pains. His treatment had been palliative and expectant.

On the 15th, he was able to walk out without his crutches. In this case there was no syphilitic history, but at times the patient had been dissipated, and probably intemperate.

Case III. Irritative cough, with pleurodynia.

May 16, 1874. Miss——, of bilio-nervous temperament, possessed, generally, a good physical condition, but had suffered severely at times from dysmenorrhæa, with cough, which distressed her about five days ago, but which had not compelled a cessation from her daily duties.

On the 14th, she began to feel extreme soreness across the chest, attended with slight hoarseness. The morning before, she began to feel a severe pain in the left sub-axillary region, aggravated by pressure or motion, with the subsidence of the general chest soreness, which had yielded to some remedies directed to the chylopoetic function. On auscultation, it was found that no pleural or pulmonic affection existed. The pulse was normal in frequency and volume; no fever existed, and the case plainly appeared to be one of pleurodynia, or, more properly, a rheumatism of the intercostal muscles. I prescribed an application of aqua ammonia, spirits of camphor and olive oil, with one grain of sulphate of quinia, every four hours. Alternately with this I ordered one drachm of sweet spirits of nitre and half a drachm of syrup of ipecac, and a pill, to be taken at night, containing one grain of blue mass, two grains of compound extract of colocynth, and one-fourth of a grain of the extract of nux vomica. She was instructed to remain quiet in bed.

On the 18th there was some relief, but the pain in the side was still felt on taking a full breath, or turning quickly. A liniment was added to the treatment described.

On the 21st, the pain was much relieved, but some cough remained, with sore throat. The pharyngeal mucous membrane was congested, and on each side white patches of ulceration were evident. I applied a strong solution of nitrate of silver by means of a sponge, and directed a gargle of carbolic acid, with quinine, iron and strychnine, internally, twice daily. Laxatives were employed for the bowels.

On the 1st of June, during the hot weather, the patient complained but little of the pain in her side. The soarness of the throat was rather persistent, but gave her little trouble. There has been, at times, severe cephalalgia, which readily yielded to ten-drop doses of the fluid extract of ergot, taken every two hours. No more than three doses were required at any one time to entirely relieve this symptom.

On the 12th, she complained of some pain and uneasiness in the side, which was attributable to a change in the weather.

By the 10th, there had been an unusual hot period for a week or more, especially during the first hours of the night, which rendered sleep difficult. After a series of showers for three days, a cool breeze sprang up from the north-west, which, while it was refreshing, endangered a relapse of rheumatic pains in those who had suffered during the spring months.

Rheumatism, although very prevalent in this locality during winter and spring, has been of much more frequent occurrence since January last.

The above cases are reported in illustration of the constitutional disturbance attending the disease. Cases I and III are those of persons whose surroundings were favorable to health; that is, necessity did not compel any undue exposure to cold or damp. The spring of 1873 was later than usual, but not more so than that of the present year. Among the colored population, and the poorer class of whites, there has been a prevalence of rheumatism and neuralgia, almost unprecedented in the history of this region.

Among adult females there has been an unusual suppression of the menses, induced by exposure to the wet and chilly weather. Two deaths occurred during the spring, of strong adult negroes, from cardiac disease following rheumatic attacks. One, a man over fifty years old, after complaining of pain in the knees and joints for some time, was suddenly attacked with pericarditis, followed by effusion. Anasarca soon succeeded, and the patient survived eleven days. The other lived but a few hours.

June, 1874.

